# Impact of Micro Silica Filler Particle Size on Mechanical Properties of Polymeric Based Composite Material

**DOI:** 10.3390/polym14224830

**Published:** 2022-11-09

**Authors:** Sidra Siraj, Ali H. Al-Marzouqi, Muhammad Z. Iqbal, Waleed Ahmed

**Affiliations:** 1 Chemical Engineering Department, College of Engineering, United Arab Emirates University, Al Ain 15551, United Arab Emirates; 2 Engineering Requirements Unit (ERU), College of Engineering, United Arab Emirates University, Al Ain 15551, United Arab Emirates

**Keywords:** sand, silica, HDPE, composite, polymer, sheets

## Abstract

In this study, silica in the form of raw local natural sand was added to high-density-polyethylene (HDPE) in order to develop a composite material in the form of sheets that could have potential applications in thin film industries, such as packaging, or recycling industries, such as in 3D printing. The silica/HDPE composite sheets were developed using a melt extruder followed by using a hot press for compression molding. The impact of two different particle sizes (25 µm and 5 µm) of the silica particles on selected properties such as toughness, elastic modulus, ductility, and composite density were analyzed. A considerable increase in the toughness and elastic modulus was observed from 0 wt% to 20 wt% with a 25 µm filler size. However, a general decreasing trend was observed in the material’s toughness and elastic modulus with decreasing particle size. A similar trend was observed for the ductility and the tensile strength of the sheets prepared from both filler particle sizes. In terms of the composite density, as the filler was increased from 20 wt% to 50 wt%, an increase in the composite densities was noticed for both particle sizes. Additionally, the sheets developed with 25 µm particle size had a slightly higher density than the 5 µm particle size, which is expected as the size can account for the higher weight. Results from this work aim to analyze the use of local sand as a filler material that can contribute towards maximizing the potential of such composite materials developed in extrusion industries.

## 1. Introduction

Over the past century, the use of plastics has been on the rise globally and is becoming alarming due to its disposal techniques that are damaging the environment, and at this current rate of usage, it is estimated that the earth will hold over 3 billion tons of plastic in the next 30 years [1]. Furthermore, it is approximated that over 50% of all the current plastic consumed ends up in nature, whether in landfills or the ocean [2]. Additionally, the waste from plastics is reported to be harmful to living organisms as their pigmentation consists of toxic and hazardous trace elements if left in the environment for long [3].

Researchers globally are developing solutions to utilize plastics in a more efficient way to incorporate them in a variety of industries for a prolonged duration of time to sustain their consumption.

Polymers are widely used in industrial applications ranging from automotive, to petroleum, to medicine, and even upcoming industries such as 3D printing [4,5,6,7,8,9,10,11]. One of the common types of polymers that is widely used in sheet-type applications is polyethylene, which includes all its variants such as HDPE, low-density polyethylene (LDPE), linear low-density polyethylene (LLDPE), etc. These polymeric materials exhibit higher mechanical strength and greater resistance to environmental stresses than other polymers [12]. These long-chained polymeric plastics are relatively easy to mold and manufacture and provide a cost-effective base polymer to start with good overall qualities [3].

The blown-film process or extrusion is the key technique typically used in fabricating thin sheets or films for packaging applications, food packaging, trashcan liners, merchandise packaging, etc. One of the critical features of any packaging material is its durability and high stability. Another key feature for thin packaging material is its high strength. However, incorporating additives and additional components is preferred to lower the use of developing completely plastic-based products. To tackle this issue, it is reported that the use of inorganic fillers is employed as it enhances the thermal and mechanical properties of the polymer as well as their durability [13,14,15,16,17]. Studies report that minerals, fillers, and hard particles increase the overall dimensional stability of the polymers [18]. Significant improvements, particularly in the mechanical properties, can be observed with increasing filler additions of inorganic exfoliated clay minerals in polymer matrices, including layered silicate compounds [19]. Studies report that the addition of small-size mineral particles with smooth spherical surfaces can result in increased matrix compactness that can lead to higher friction bonding [20].

Additionally, the elastic modulus is a property that can be easily enhanced by adding micro- or nanoscale particles since the particles have a higher stiffness than the polymeric matrices. The applied stress can be easily transferred from the polymer matrix to the particles, enhancing the material’s tensile strength [19,21]. Studies report that the addition of hard particles in polymers, such as in polyethylene, resulted in improved impact toughness as well [22,23,24]. Moreover, it can be said that the addition of particles has an effect at smaller stress levels. For instance, the plastic deformation can be distributed by the cavitation caused by adding fillers, which allows the polymer to yield. This results in extended areas of plastic deformation and, thus, increases the amount of energy that can be absorbed by the composite material, thereby improving the overall toughness [22]

Mineral fillers such as calcium carbonate (CaCO_3_), titanium dioxide (TiO_2_), silicon dioxide (SiO_2_), etc., are widely used to develop polymer composite materials [25,26,27,28,29,30,31]. Combinations of several minerals to develop hybrid composites have also contributed to improved mechanical properties. Studies report that the element detected the most is considered the first primary phase, which mainly contributes and impacts the properties [15]. However, if the same filler with the same combination of mineral content is added, mechanical comparisons can be performed on composites with varying filler additions [32]. It is to be noted that the mechanical properties of a polymer-composite material depend strongly on key factors, such as the particle size of the filler, the interface adhesion between the particle and the matrix, and the particle loading [19]. Adding fillers can even enhance the tight packing of the polymer matrices; for instance, smaller particles can provide higher strength and increase the compactness of a polymer matrix Additionally, fillers can appear spherical but be present in clusters rather than individual particles due to interparticle interactions. The particles can vary anywhere from 0.1 µm up to 1000 µm depending on the application type and properties desired at the micro scale [19,20,33].

Overall, composites developed with fillers are reported to exhibit several advantages over conventional materials or their individual components, for instance providing key benefits in bulk weight and costs [34]. Inorganic fillers such as silica are also considered because they fulfil the purpose of enhancing mechanical properties such as strength and durability and, at the same time, are safe for human health; therefore, they can be incorporated into products that involve human interaction, such as packaging or even in building construction materials [35].

Due to its unreactive nature, high thermal stability, and high durability, silica is one of the most widely chosen fillers in a variety of fields such as electronics, textiles, construction, 3D printing, etc. [10,36,37,38,39,40,41,42]. It is also reported that the surface of precipitated or hydrated silica is hydrophilic due to the presence of a high number of silanol groups that can bond and adhere through the presence of hydrogen bonding [43]. Studies also show the incorporation of sand into polymers in building materials, recycling, and managing wastes [44,45,46,47,48]. Furthermore, silica can be easily surface-modified as per the need of an application to increase or decrease the hydrophilicity, making it a very versatile mineral to use as a filler material [49,50]. For instance, studies report that hydrophobic silica has the ability to double the adhesion of binders in contrast to hydrophilic silica, which halves it [51]. Moreover, modifying silica particles’ surface hydrophobicity leads to improved overall compound stability [52]. This gives the ability to finely tune the interparticle interactions, which can modify the way the particles assemble under applied stress [53]. Silica and its derivates are also expected to self-aggregate and form a network of particles within the matrix, increasing the formation of links and resulting in an overall integrated structure [54].

Furthermore, geographically speaking, the middle east has a vast abundance of areas with raw sand whose main component is usually silica. Since variants of silica are extensively used as fillers to improve the properties of composite materials, the consideration of using local raw sand by itself was decided to be studied. Additionally, in terms of polymers, since HDPE is widely used as a large-scale commodity polymer for thin film products [36,55], and due to its ease of availability, recyclability [56], good mechanical strength, high chemical resistance, and low cost [54,57], HDPE was considered as the choice of polymer for this study. HDPE is very light in weight, is water-resistant, and can be easily recycled, making it a good choice for packaging applications. HDPE also has good impact resistance, as well as has shown good resistance to insects, molds, and mildew. Fillers can enhance several properties of HDPE, for instance, strength, modulus, toughness, hardness, and durability, as well as improve thermal conductivity [57,58]. The addition of fillers to HDPE also helps prevents shrinkage of the material and is also reported to adsorb high-energy photons when exposed to UV light, prolonging its ability to extended outdoor exposure [59,60].

Moreover, since in thin film applications, extrusion is considered the major technique [61], a lab-scale twin-screw extruder setup is a great bench-scale option to be considered for developing such composites employed in this study [62].

There is a significant rise globally in the use or reuse of natural items and industrial by-products as raw materials or additional supplementary materials in order to maximize material usage and potential and reduce their negative environmental and economic impacts. Since these by-products or raw materials are abundant, it is smart to incorporate them into products to reduce the ecological and economic burden caused due to an increase in plastics and limited natural resources [20]. Several industries also prefer opting for low-cost reinforcements that can improve the properties of the material and, at the same time, also take into consideration how easily the material can be accessed and manufactured. Additionally, the already existing industrial-scale processing techniques, such as casting, extrusion, or compression, are an added advantage as they involve low fixed capital and production costs to utilize these materials [15]. Moreover, according to a study conducted for a silica based composite material, one of the key mechanical properties, i.e., the elastic modulus, increased linearly with the content of the silica added and the impact of the particle size was insignificant. Since filler at a micro level is easier to access and develop, opting for a filler at the microscale to enhance properties is a more efficient and cost-effective approach to develop composite materials rather than investing in expensive technology to obtain finer particles that essentially will perform a similar job at the nanoscale with a higher loading [19]. Another study highlights the comparison of values of Young’s modulus between micro silica and nano-silica, which demonstrated values higher in the micro case up to a certain filler weight loading [21]. Another study suggested that filler loading is the determining property for mechanical properties, for instance, for the elastic modulus. In contrast, other filler characteristics such as shape and size are secondary fine-tuning factors that contribute towards altering the properties of the material [63]. Additionally, research also suggests that it is difficult to recognize the effect of filler size and shape on the mechanical property of composites [64]. Another property to keenly monitor with smaller particles is their high surface-free energy. When the interparticle forces exceed the particulate mass, agglomerations can easily occur and this may contribute negatively by reducing the polymer–filler interactions [63]. It all comes down to the desired outcome one requires for their developed material, and how much time, technology, and energy one is willing to invest to obtain the filler size they are satisfied with that gives them acceptable results.

The results from this study will explore the possibility of using raw sand (silica) in sheets that could be used in packaging applications. Therefore, the key objective of this study was to incorporate local sand as a filler material to develop composite sheets and study the impact of particle size on some of the mechano-physical properties of the developed material. The novelty of this study lies in the efforts and contributions made towards studying the potential of using local sand as a filler particle that can be of great interest to further research in this geographical area. The aim here is to develop simple yet effective solutions focused on utilizing local resources that simultaneously can be focused on utilizing local resources and contributing towards developing sustainable products that can lower ecological and economical burdens. Such a study can open pathways to develop innovations focused on sustainable materials and promote the incorporation of local resources that are abundant in nature to decrease the negative environmental impacts.

## 2. Materials and Methods

The sand (mainly in the form of silica) was collected from local areas of the country. The obtained sand was handpicked to remove any more oversized items and sieved through a 25 microns aperture sieve (200 mm diameter, stainless steel mesh) to obtain 25 µm size of particles and further ground using a planetary ball mill (PL-400, Retsch, Haan, Germany) to obtain 5 µm size of particles. The composition of the local sand was reported to be 47 wt% silicates, with 26 wt% carbonates and approximately 14 wt% quartz. Additionally, the mineral component of SiO_2_ was reported to be approximately 37 wt% [55]. HDPE pellets were purchased from Sigma-Aldrich, Saint Louis, MO, USA (Density = 0.98 g/cm^3^, Mw~125,000 g/mol, melt flow index 2.2 g/10 min, shear rate 0.004 s^−1^) [65,66,67,68].

**Figure 1:**
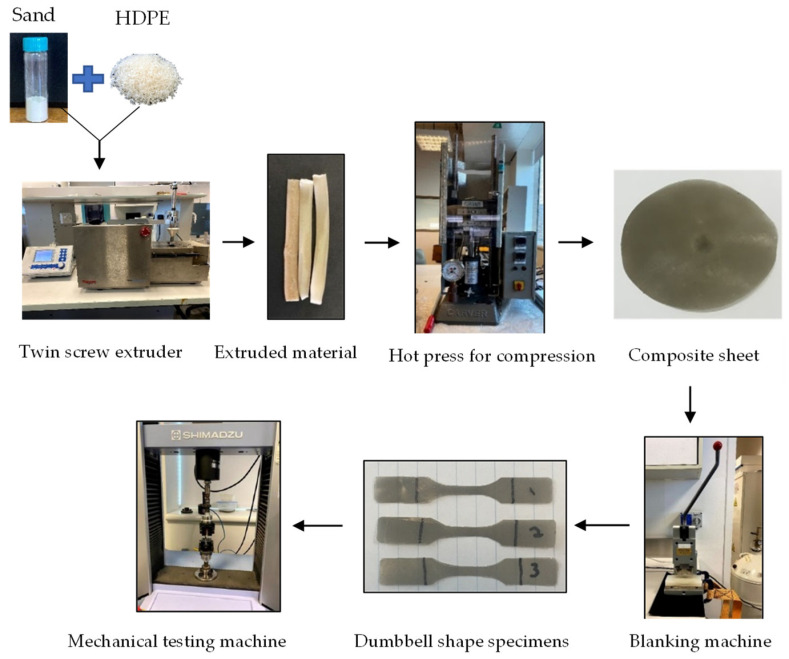
Experimental process.

The two sets of particle sizes were added to the HDPE pellets in a chosen set of weight ratios (20, 35, and 50 wt%). The mix was added into a twin-screw melt-blend extruder (MiniLab HAAKE Rheomex CTW5, Karlsruhe, Germany). The closed loop cycle followed a set temperature of 170 °C, 15 min at an rpm of 100. These parameters were kept constant through all the ratios. A similar run was also performed for pure HDPE pellets [69,70,71,72]. The total feed to the extruder was kept constant at 4 g as required by the setup [8,55]. Once the run was complete, the composite material was collected as the extruded material from the exit valve of the setup. The material was chopped into small sizes of 1 g and placed into the hot press for compression molding to obtain the composite sheets. The hot press (Carver’s press (Carver^TM^ Lab Presses)) was operated under 5000 psi and at the same extrusion temperature for approximately 10 min. All the processing conditions were chosen after following literature reviews for similar systems and successful results from trial and error [12,22,73]. The only variables that would be changed in this experiment are the particle size and the filler loading. The sheets were then cut into dumbbell-shaped mechanical testing specimens using a blanking machine to analyze their toughness, elastic modulus, and ductility properties. Figure 1 presents the process, Table 1 presents chosen weight percentages for the preparation of the sand/polymer composite sheets, and the experimental conditions are illustrated in Table 2. Additionally, the data in Table 1 and Table 2 are also represented in a simple table format for the ease of any researcher trying to replicate the experiment and for the ease of anyone only referring to the amounts needed while experimenting for such a system of composite material.

Table 3 shows images of sand/polymer composite sheets prepared from 25 µm sand particles and 5 µm sand particles, respectively. All the sheets were roughly 100 mm in diameter. The sheets were inspected visually. It was evident that increasing the filler led to a darker appearance of the sheets. Additionally, it could also be seen that the dispersion of the particles in the composite sheets was quite random, and an increase in the non-homogeneity was observed with increasing particle wt% for both sets of particle sizes.

### 2.1. Characterization

#### 2.1.1. Scanning Electron Microscope (SEM)

A JEOL/EO scanning electron microscope (SEM) with a spot size of 40 was operated at 2 kV to image the particles of sand measuring 25 µm and 5 µm at their surface. Using a vacuum sputter coater, samples were coated with Au/C to improve the image’s conductivity and quality. Similarly, SEM analysis was used to observe the surface morphology of the selected composite sheets as well as neat HDPE sheets. Using double-sided carbon tape, the selected composite sheets were placed on an aluminum pin-mount adapter and then using a sputter-coater, they were coated with gold to avoid any electrostatic charging during the examination. A high vacuum mode with an acceleration voltage of 15 kV was used and the images were obtained.

#### 2.1.2. Mechanical Properties

**Figure 2:**
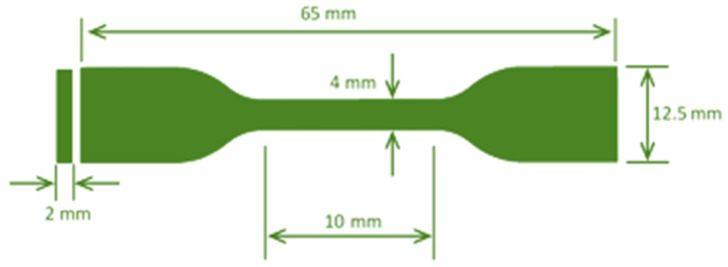
Sample dimensions.

The universal testing machine (UTM) determined the composite sheets’ mechanical properties using the American Society for Testing and Materials (ASTM)-D 638. Dumbbell-shaped test specimens (as seen in Figure 2) were prepared using a blanking machine. Slight variations in sample thicknesses were observed, which is attributed directly to the processing itself [3]. The study was conducted on a Zwick 50 kN. The crosshead motion rate was set to 100 mm/min, which was taken from the ASTM D 790 standard. Replicates of each sample test were performed for increased accuracy. The sample thickness was measured at several locations along the gage length, and the samples with consistent and similar values were selected for further analysis [74]. All dimensions (i.e., the length (l), width (w), and thickness (t)) of the samples are tabulated in Table 4.

One of the mechanical properties that is of great significance is the toughness of a material. It indicates the amount of energy the material can absorb on an impact, and this value can be estimated by determining the area under the stress-strain curve. Using the following Equation (1):

(1) Toughness [MPa]=σ×ε

where σ is the stress in MPa and ε is the strain.

Additionally, the elastic modulus for the developed composite material was analyzed by examining the data obtained from the linear part of their corresponding stress-strain plots. The elastic modulus is the stiffness or the ratio between the stress–strain plot of the material undergoing elastic deformation in a tensile test [19]. The modulus of elasticity was estimated by using the average initial cross-sectional area concerning the gage length of the sample. Equation (2) represents how the elastic modulus can be calculated.

(2) $$\;E\;\left[MPa\right]=\frac{F\times L_{o\;}}{A\times ∆L}$$

where *E* is the elastic modulus in *MPa*, *F* is the force exerted on the sample under tension in N, *L_o_* is the original gage length of the sample in mm, *A* represents the original cross-sectional area of the sample material in mm^2^, and ∆*L* is the change in length of the sample in mm.

When the uniform deformation for gage length is a property of consideration, measuring elongation is quite helpful. This property quantifies the importance of good engineering design. Additionally, the elongation at break (ductility) can be prevented by observing the extension of the sample at the final breakpoint. Ductility can be calculated using the following Equation (3):

(3) $$\mathrm{Ductility}\;\left(\%\right)=\frac{L_{f}-L_{o\;}}{L_{o}}\times 100$$

where *L_o_* is the original gage length in mm, and *L_f_* is the final length at the break in mm.

#### 2.1.3. Composite Density

In order to calculate the dry density of the filler, ASTM D7263-09 was followed [75]. A controlled drying process in an electric drying oven was used to dry the sand. Using the following Equation (4), the density of the sand was calculated [76].

(4) $$\;\;\;\;\;\;\;\;\;\;\;\;\rho_{\mathrm{filler}}=\frac{m_{\mathrm{filler}}}{Volume}$$

where *ρ*_filler_ is the density in kg/m^3^ of the filler particle, *m*_filler_ is the mass in kg of the filler particle and *Volume* is the measured cylinder used in m^3^

In order to calculate the apparent densities of the polymers, ASTM D792-20 was followed [75]. The masses of the samples were measured in air and water and using this data, the apparent densities can be estimated using the following Equation (5):

(5) $$\rho_{\mathrm{pol}}=\frac{mass_{\mathrm{air}}\;\rho_{\mathrm{water}}}{mass_{\mathrm{air}}+mass_{\mathrm{water}}}$$

where *ρ*_pol_ is the apparent density in kg/m^3^ of the polymer, *mass*_air_ is the mass in kg of the measured sample in air, *mass*_water_ is the mass in kg of the measured sample in water, and *ρ*_water_ is the density in kg/m^3^ of water.

Next, the linear rule of mixing assumes that the volume-weighted average of the matrix and the dispersed phases can account for the composite material’s theoretical density. The equation also assumes that the filler does not affect the polymer matrix in any crystal formation nor is there any air in the composite material. The density of the composite material can simply be defined as the ratio of the densities of the filler and polymer to the total volume, which is represented by the sum of the products of the filler and polymer densities and their corresponding masses [77].The equation for composite density is represented in Equation (6):

(6) $$\rho_{\mathrm{composite}}=\frac{\rho_{f}\;\rho_{m}}{\rho_{m}\;m_{f\;}+\rho_{f}\;(1-m_{f})}$$

where *ρ*_composite_ is the density in kg/m^3^ of the composite material, *ρ*_f_ is the density in kg/m^3^ of the sand (silica filler), *ρ*_m_ is the density of the polymer matrix (HDPE) in kg/m^3^, and *m*_f_ is the mass fraction of the filler used.

The samples were cut as per ASTM D6287-17 to measure their volumes [78], and the thickness of the samples was measured using a thickness gauge (model 547–526S, Mitutoyo, Kawasaki, Japan, resolution 0.001 mm) along several points to ensure uniformity, First, their weights were measured using a weighing balance (Citizen-CX 220, d 0.001 g, ManualsLib, Bangalore, India) for the experimental densities of the developed composite. These values were used to estimate the experiential densities of the composite material by calculating their respective mass-to-volume ratios.

## 3. Results and Discussion

### 3.1. Morphology

SEM analysis was conducted to estimate the particle sizes and shapes of the local raw sand used as the filler in the experiment. Table 5 represents the SEM images of sand filler of both 25 µm and 5 µm particle size as selected sheets prepared, respectively.

The images illustrate that the 25 µm particle has a higher inter-particle distance than those in 5 µm particles. Additionally, the 25 µm are larger and more irregular in size compared to the 5 µm particle size, which is smaller and finer in appearance. A few sheets were selected to observe the morphology. Table 5 also shows a neat HDPE sheet (0 wt%) and selected 35 wt% particle size composite sheets prepared from 25 µm and 5 µm particles, respectively. The pure HDPE sheet shows a smooth structure with an absence of any particles in it. The outcome of the variation in the particle size as fillers in the composite sheets can be well observed as in the case of 25 µm, the particles are much further apart, whereas in the case of 5 µm, the particles appear closer together. However, it is to be noted that a decreased inter-particle distance can led to agglomeration formation, which can lead to material with a brittle nature [36].

### 3.2. Mechanical Properties

The impact of the filler particle size was assessed on selected mechanical properties such as toughness, elastic modulus, and ductility of the prepared composite sheets.

**Figure 3:**
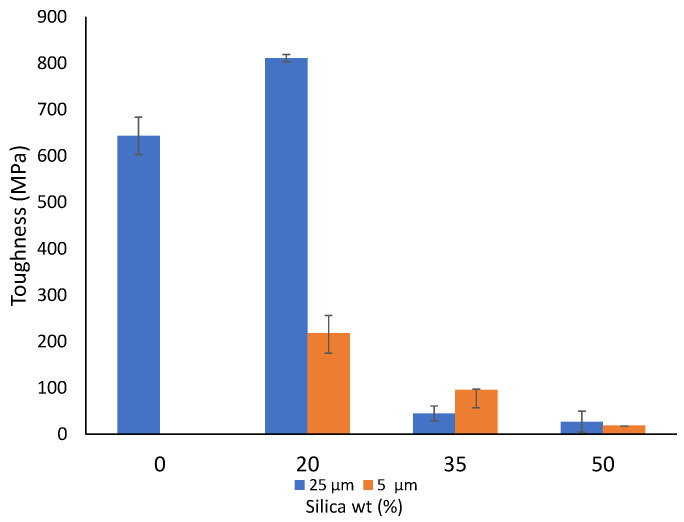
Toughness of the prepared composite sheets.

Toughness is associated with the energy a material can absorb in an impact, which can be a critical value to monitor based on the type of application. Figure 3 represents the toughness value for both 25 µm and 5 µm filler sizes. It can be seen that the toughness value experiences a sharp increase from around 611 MPa at 0 wt% to 811 MPa at 20 wt% in the case of 25 µm. Beyond this point, the toughness starts to decrease. Such a substantial increase is noted in some other composite studies as well [79], and it can be associated with the adsorption of silica clusters to the polymeric chains [80,81] and due to the silica particles promoting partial stress within the matrix, which can change the direction of crack development [82,83,84].

Furthermore, as the filler wt% was increased, the toughness decreased for sheets prepared from the 5 µm filler size. The decrease in these values can be associated with the agglomerate formation, which leads to structural instability of the material, thereby reducing the ability of the composite to have a higher absorption of energy at impact, leading to the formation of cracks at the surface and eventually breaking. Many researchers add modifying agents to increase the impact resistance of materials [85,86]. However, it is noteworthy that at 35 wt%, the toughness of the 5 µm particle is higher than the 25 µm particle. This can probably be based on how the particles have arranged themselves in the matrix. Due to 25 µm being a larger size, the random dispersion of bigger particles could have created more stress concentrations than in the case of smaller-sized particles, leading to such a possibility.

**Figure 4:**
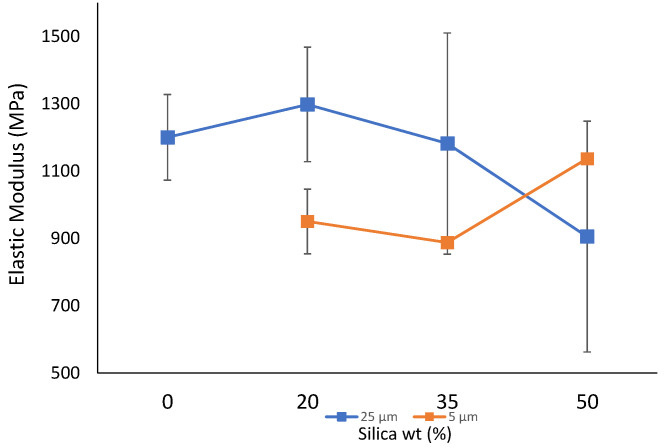
Elastic modulus of the prepared composite sheets.

Furthermore, it was observed that an increase in filler addition from 0 wt% to 20 wt% increased the elastic modulus from 1201 MPa to 1298 MPa for a filler size of 25 µm, which is comparable to the literature values [1,87,88]. Beyond this point, a gradual decrease was observed. For the composite material developed with a 5 µm filler size, a similar decreasing trend was observed with the exception at 50 wt%. Moreover, as a comparison, it could be said that all the composite sheets prepared from 5 µm filler particles resulted in a lower elastic modulus value than those prepared from 25 µm particles with an exception at 50 wt%. The decrease in values can be associated with the development of stress concentrations weakening the matrix. Additionally, the formation of hydrogen bonding can also propagate cracks at weak interfaces, which can facilitate the brittle nature of the sheets, leading to failure [56,87,89]. Moreover, the possible variation at a higher wt% could be due to extensive molecular orientation that could occur in thermoplastic-based films, along with random dispersion of the filler particles leading to inconsistent results [1]. Figure 4 represents the elastic modulus for composite sheets prepared with both sets of filler particles.

**Figure 5:**
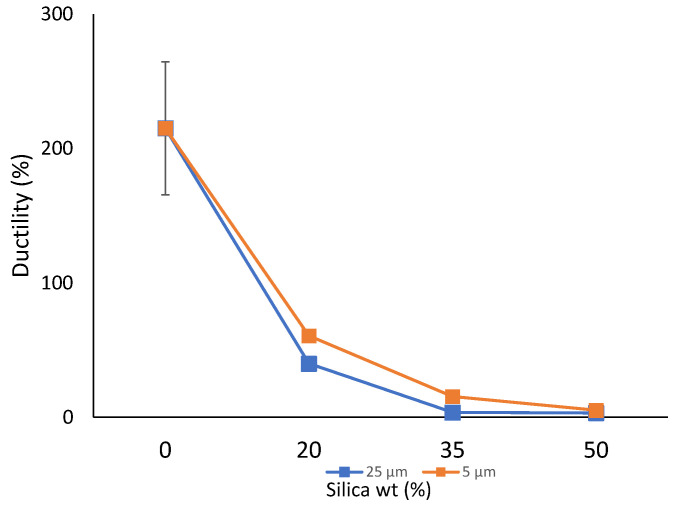
Ductility of the prepared composite sheets.

The strain at the point of failure can be better understood by studying the material’s ductility. A material’s ability to deform plastically and adapt to the load applied can be of great value in several industrial applications requiring highly flexible materials. Figure 5 represents the ductility of all the prepared composite sheets. The pure HDPE sheet had a high ductility of over 150%, which is well reported [90,91]. Almost similar decreasing trends in the ductility were observed with an increase in filler addition from 0 wt% to 50 wt% for sheets prepared from both particle sizes. Additionally, it can be said that a substantial decrease in the ductility (up to 98%) was noted for both 25 µm and 5 µm filler sizes from 0 wt% to 50 wt%. This degrading behavior can possibly be linked to unexfoliated aggregate development and the formation of structural voids, which reduces the matrix to show improved ductility. This kind of behavior is also noted in other polymer-composite studies [85,92]. The literature reports that the addition of particles and aggregates delays the initiation of cracks and inhibits the steady-state flat crack propagation resulting in a loss of the ductility of the material. Additionally, particles larger than the fiber spacing contribute to the balling of fibers, resulting in poor dispersion and lowering the property [20].

**Figure 6:**
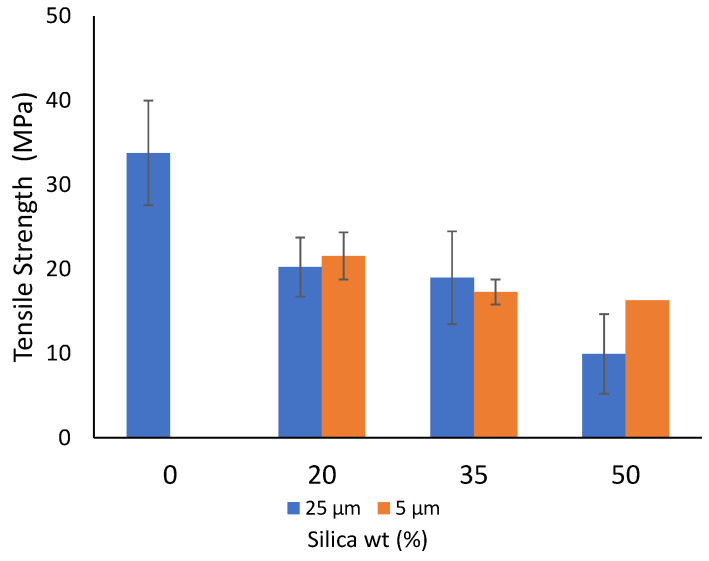
Tensile strength of the prepared composite sheets.

Figure 6 represents the effect of varying filler concentrations and particle size on the tensile strength of the developed composites. In general, a decrease in the tensile strength was noticed for the composite material developed from both 25 µm and 5 µm size filler particles. For instance, for the composite developed from 25 µm particles, the tensile strength decreased from approximately 20 MPa at 10 wt% to 10 MPa at 50 wt%, whereas with the composites prepared from 5 µm particles, the value decreased from 22 MPa at 10 wt% to 16 MPa at 50 wt%. Such a decreasing trend can be explained by the particles randomly arranging themselves in a way that limits the stress transfer [93]. Not much difference was observed for the values at 20 wt% and 25 wt% at both filler sizes. However, a large variation in properties, for instance, at 50 wt% is expected to occur in thermoplastic-based thin films undergoing extensive molecular orientation [93]. Moreover, the decreased tensile strength also causes increased brittleness, and this can be attributed to the formation of agglomerates. These agglomerates form initiation spots of stress concentrations that lead to failure. Moreover, the formation of voids in matrices is also reported to contribute towards decreased strength values [94,95]. All the mechanical data for all the prepared composite sheets are tabulated in Table 6.

### 3.3. Composite Density

**Figure 7:**
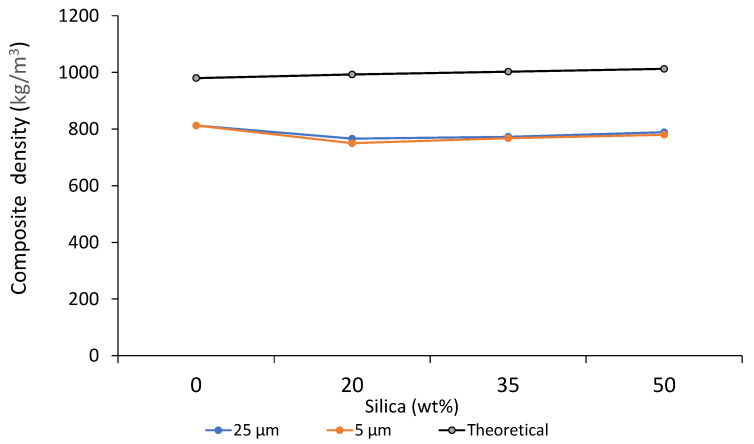
Composite density.

In general, the experimental composite density experienced a decrease from 0 wt% to 20 wt% in both the cases of composite sheets prepared from 25 µm and 5 µm particles, respectively. This could be due to the matrix’s random filler dispersion of the introduced filler particles. Additionally, since the sand particles’ density (1047.53 kg/m^3^) [55] is slightly higher than that of the polymer, it could have impacted the calculated value. Beyond this point, a slight increase was noted with an increase in filler addition. In the case of 25 µm particles, that density varied from 766 kg/m^3^ at 20 wt% to 789 kg/m^3^ at 50 wt%. For the composite sheets prepared from 5 µm particles, the density ranged from 750 kg/m^3^ to 780 kg/m^3^. In all cases, the density of the sheets prepared from 25 µm particles was more than its corresponding sheets prepared from 5 µm particles. These representations can be seen in Figure 7. This can be possibly explained by the fact that the 25 µm particles are bigger in size and nature than the 5 µm particles, thereby contributing to a greater density value. Furthermore, both cases obtained significantly lower values than theoretical values. For instance, a decrease of up to 23% was observed for sheets prepared from 25 µm particles and close to 25% for the sheets prepared from 25 µm particles at 20 wt% filler addition. Such high variations can be associated with the high temperatures applied during the processing of the sample material. Moreover, residual moisture can be degassed with the sample material due to inadequate and non-homogeneous mixing, which can also contribute to the lowered density value as the polymer matrix attains high viscosity [96,97]. In industrial applications requiring materials to have a lower density, for instance, applications requiring floating, fillers can be a great way to lower this property [98]. Furthermore, handling the materials can benefit from it as lighter materials can be easier to transport, thereby reducing cost [99]. Table 7 reports the composite density data for all the prepared composite sheets.

## 4. Conclusions

This study developed composite sheets using silica microparticles of two different sizes as a filler in a HDPE polymeric matrix. This was analyzed on the mechanical properties and composite densities. In general, the majority of the mechanical characterization showed a decrease in their values with the filler addition. However, a notable increase in the toughness and elastic modulus of the composite material was observed with 20 wt% filler at 25 µm particle size, promoting that at this particle size and wt%, enhanced properties were obtained. As for the ductility of the material, a sharp decrease was observed in both cases of particle size, implying the idea that the filler addition led to agglomerates that resulted in several areas of stress concentrations, leading to such lowered values, which is expected in composite materials. In terms of the composite densities, increasing the filler particles at both particle sizes led to a decrease in the density value from 0 wt% to 20 wt%, and this can be considered an enhanced property, depending on the type of application, for instance, when transportation is considered. Further studies on silica with polymers are underway to understand its potential to be used as a filler material in thin film applications. The data from this research promote the promising use of local sand as a filler material that can enhance composites’ mechanical and density properties and open pathways to conduct more studies to explore suitable applications for such an abundant filler. Future work is recommended to be directed towards studying and analyzing the rheology of the developed composite materials, as rheological properties can further help in understanding the microscopic and mesoscopic level of structures within the matrix as the filler dispersion and filler–matrix interactions are highly linked to the viscoelastic properties of the material. Additionally, the impact of potential additives such as coupling agents and compatibilizers that can improve the interfacial adhesion in the composite material, which can further enhance the mechanical properties, must be explored. Moreover, this research can further be extended by extensively studying the hydrophobization of the surface of the silica filler particles and the evaluation of the impact on the nature of the surface of the filler particle and the influence on the properties of the developed composite material [100,101]. Future studies can also be aimed at utilizing recycled HDPE to lower the ecological impact further and promote an even more sustainable approach to developing such a composite material. The potential to recycle such a product can invite scientific and business interests that could be vital to contributing towards global economic strategy [15,47,68,102,103,104]. This study aimed to highlight the potential of using a local resource and test its feasibility to be used as a useful material in developing an innovative product that can be easily commercialized with further development.

## Figures and Tables

**Table 1:** Chosen weight percentages for sand, and HDPE.

	Sample	Sand (wt%)	HDPE (wt%)
Sand/polymer composite sheets prepared from 25 µm and 5 µm sand particles	0 wt%	0	100
	20 wt%	20	80
	35 wt%	35	65
	50 wt%	50	50

**Table 2:** Experimental conditions.

Parameters						
	Temperature (°C)	Time (min)	Particle Size (µm)	Total Input (g)	Screw Speed (rpm)	Pressure (psi)
Melt extrusion	170	15	25 and 5	4	100	-
Hot press compression	170	10	25 and 5	1	-	5000

**Table 3:** Prepared composite sheets with varying filler wt%.

	0 wt%	20 wt%	35 wt%	50 wt%
Sand/polymer composite sheets prepared from 25 µm	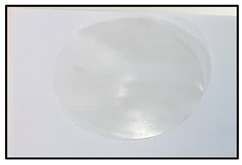	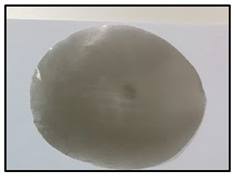	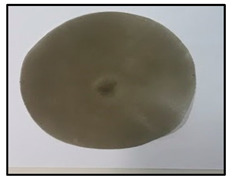	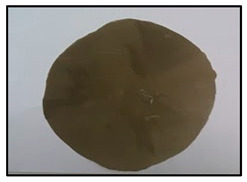
Sand/polymer composite sheets prepared from 5 µm		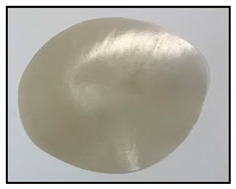	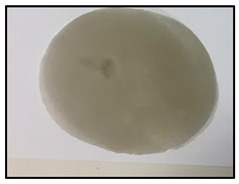	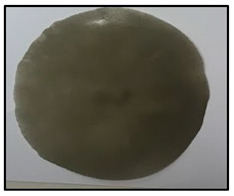

**Table 4:** Dimensions of prepared composite sheets.

Sample		Dimensions (l × w × t)
Sand/polymer composite sheets prepared from 25 µm sand particles	0 wt%	10 × 4 × 0.62
	20 wt%	10 × 4 × 0.41
	35 wt%	10 × 4 × 0.45
	50 wt%	10 × 4 × 0.57
Sand/polymer composite sheets prepared from 5 µm sand particles	20 wt%	10 × 4 × 0.33
	35 wt%	10 × 4 × 0.44
	50 wt%	10 × 4 × 0.53

**Table 5:** SEM images of 25 µm, 5 µm, 0 wt%, and 35 wt% were prepared from both particle sizes.

**25 µm**	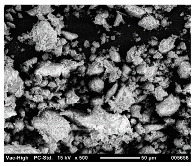	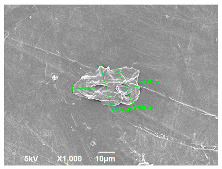	
**5 µm**	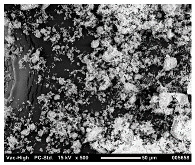	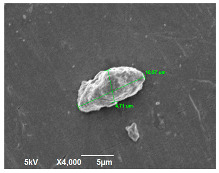	
**Sheets**	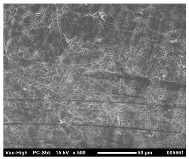 Pure HDPE	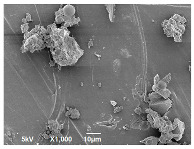 35 wt%, 25 µm	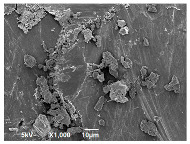 35 wt%, 5 µm

**Table 6:** Mechanical properties of the composite sheets.

Sample		Toughness (MPa)	Elastic Modulus (MPa)	Ductility (%)	Tensile Strength (MPa)
	0 wt%	643.5 ± 40.3	1200.77 ± 127.3	215 ± 49.5	33.76 ± 6.2
Sand/polymer composite sheets prepared from 25 µm sand particles	20 wt%	810.75 ± 8.1	1298.33 ± 169.8	29.67 ± 1.41	20.23 ± 3.5
	35 wt%	44.80 ± 15.9	1182.33 ± 328.4	3.7 ± 0.72	18.96 ± 5.5
	50 wt%	26.85 ± 22.7	905.72 ± 343.1	3.25 ± 1.34	9.93 ± 4.7
Sand/polymer composite sheets prepared from 5 µm sand particles	20 wt%	217.33 ± 42.4	950.59 ± 86.6	20.67 ± 0.58	21.56 ± 0.6
	35 wt%	95.33 ± 38.6	887.47 ± 96.2	11.87 ± 4.51	17.28 ± 2.8
	50 wt%	18.85 ± 1.6	1137.05 ± 8.2	2.07 ± 0.25	16.3 ± 1.48

**Table 7:** Composite density of the composite sheets.

Sample	Composite Density Theoretical (kg/m^3^)	Composite Density for Sheets with 25 µm (kg/m^3^)	Composite Density for Sheets with 5 µm (kg/m^3^)
0 wt%	980.00	812.5	812.5
20 wt%	992.80	766.14	750.0
35 wt%	1002.62	772.92	767.71
50 wt%	1012.64	788.82	780.0

## Data Availability

Not applicable.
